# Impact of pituitary pars intermedia dysfunction on inflammation within the equine reproductive tract of the mare

**DOI:** 10.3389/fvets.2026.1758501

**Published:** 2026-03-26

**Authors:** Jocelyn Howard, Isabella Hamner, Rebecca A. Crook, Cheyenne Elliott, Elaine Carnevale, Stephen J. Coleman, Brody A. Klinglesmith, Patrick M. McCue, Jenny L. Sones, Carleigh E. Fedorka

**Affiliations:** 1 Department of Animal Sciences, Colorado State University, Fort Collins, CO, United States; 2 Department of Biomedical Sciences, Colorado State University, Fort Collins, CO, United States; 3 Department of Clinical Sciences, Colorado State University, Fort Collins, CO, United States

**Keywords:** endometrium, equine, follicular fluid, inflammation, PPID

## Abstract

**Introduction:**

Pituitary pars intermedia dysfunction (PPID) is an age-related endocrinopathy associated with elevated systemic inflammation, and specifically an upregulation of interleukin-8 (IL-8). It is unknown if PPID in concomitant with reproductive tract inflammation. This is a pertinent question, as chronic inflammation of the endometrium and ovary would impede fertility. Therefore, the objective of this study was to evaluate the impact of PPID on the reproductive tract of the mare.

**Methods:**

PPID was diagnosed via thyrotropin releasing-hormone (TRH) stimulation test, where PPID was diagnosed as ACTH>120 pg/mL post-stimulation, and controls were diagnosed as ACTH<60 pg/mL. In the first study, seven PPID mares and four age-matched control mares had endometrial biopsies taken when in diestrus. In the second study, seven PPID mares and ten age-matched control mares had follicular fluid aspirated from preovulatory follicles using transvaginal aspirations. Analysis included qPCR analysis of select targets associated with endometrial inflammation in addition to immunochemistry for leukocytes. Finally, immunoassay was used to assess the production of systemic and follicular fluid cytokines. Statistics were performed using SAS 9.4®. The impact of PPID on the expression of transcripts, production of cytokines, and number of leukocytes was evaluated using an unequal variances t-test. The correlation between ACTH and number of leukocytes was assessed using a Pearson’s correlation test. Significance was set to *p* < 0.05, with trends noted at *p* < 0.1.

**Results:**

Only endometrial IL-8 was found to increase in expression in the PPID population (*p* = 0.02). There was a positive correlation between ACTH and the endometrial expression of IL-8 (*p* < 0.001; R^2^ = 0.80). A weak correlation was also noted between ACTH and expression of *IL-6* (*p* = 0.04; R^2^ = 0.41) and *IFNγ* (*p* < 0.01; R^2^ = 0.63). PPID mares had more endometrial leukocytes than control animals (*p* = 0.03), which was also positively correlated with ACTH (*p* = 0.03; R^2^ = 0.47). An increase in IL-8 was also noted in the follicular fluid (*p* < 0.01) of PPID mares.

**Discussion:**

The systemic inflammation previously reported in the PPID animal was also observed within the reproductive tract of the mare, and this was found as both expression and production of pro-inflammatory cytokines in addition to presence of leukocytes. Furthermore, this inflammation was noted within the uterus in addition to the preovulatory follicle. Future research is warranted to determine if this increase in inflammation of the reproductive tract is detrimental to the fertility of PPID mares.

## Graphical abstract

**Figure:**
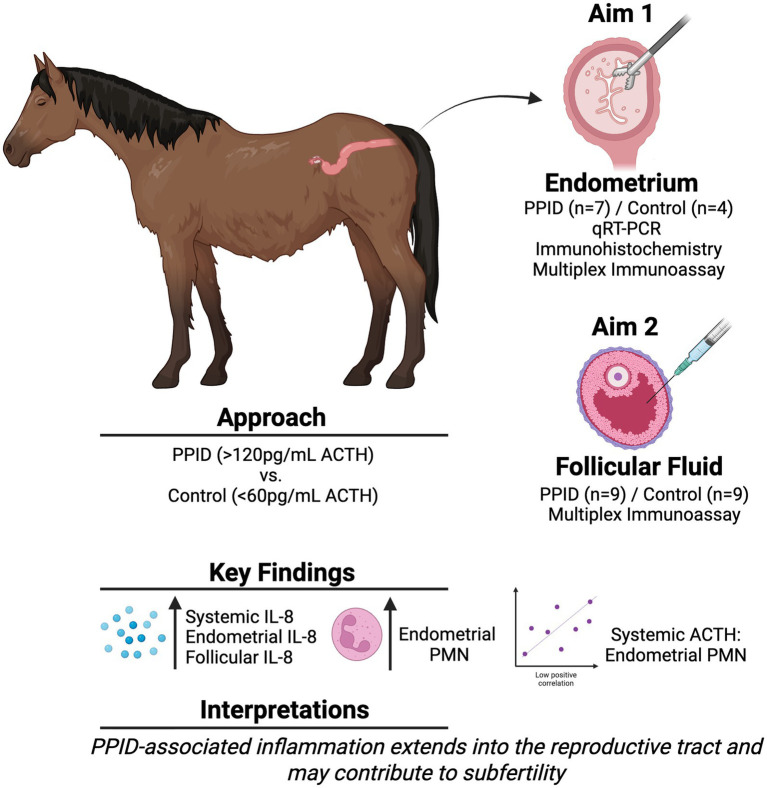
PPID-associated systemic inflammation extends into the reproductive tract of mares, resulting in elevated IL-8 across systemic, endometrial, and follicular compartments and increased endometrial leukocyte infiltration. This inflammatory environment is associated with circulating ACTH and may contribute to subfertility.

## 1 Introduction

Pituitary pars intermedia dysfunction (PPID) is a progressive neurodegenerative endocrinopathy that affects approximately 20% of aged horses (1, 2). Pituitary dysfunction associated with this disease leads to increased secretion of pro-opiomelanocortin peptide hormones, and this includes an increase in *α*-melanocyte stimulating hormone (α-MSH), *β*-endorphin, and adrenocorticotropin hormone (ACTH) (3–5). Endocrine dysfunction then leads to hirsutism, muscle atrophy, polyuria, polydipsia, and hyperhidrosis (3, 6, 7). PPID animals have been found to have elevated expression and production of the pro-inflammatory cytokine CXCL8 (7–9), which is responsible for the chemotaxis of leukocytes to sites of inflammation (10). This systemic inflammation may increase the risk of secondary infections and laminitis (11). Unfortunately, it is unknown if PPID-associated chronic inflammation is present within other body systems, including the reproductive tract.

Chronic inflammatory signaling disrupts the delicate balance of cytokines, hormones, and immune cells required for normal reproductive physiology. In the ovary, this can impair follicular development, oocyte quality, and corpus luteum function (12). Within the uterus, sustained inflammation compromises both sperm and embryo receptivity, and is a primary cause of reduced fertility in the mare (13, 14). Mares with chronic inflammation of the reproductive tract are predisposed to chronic infections (15) and increased early embryonic loss (16). Thus, while transient inflammation is necessary for reproductive success, persistent inflammation is detrimental at nearly every stage, highlighting the necessity of immune homeostasis. It is unknown if the systemic inflammation noted in PPID animals predisposes animals to chronic inflammation within the reproductive tract, and this question needs answered.

Therefore, we aimed to assess the impact of PPID on reproductive form and function. These findings combined with the knowledge that PPID causes increased inflammation within the body have led to the hypothesis that PPID is associated with inflammation within the reproductive tract of the mare. We hypothesize that the elevated inflammation noted in circulation of PPID animals will be associated with increased inflammation in the reproductive tract. An increase in pro-inflammatory production would then be hypothesized to lead to increased chemotaxis of leukocytes to endometrial lumen. Additionally, we hypothesize that this inflammation will not be isolated to the endometrium but will also be evident in follicular fluid. PPID-associated inflammation could explain the subfertility noted in this diseased population of mares, which deserves considerable attention.

## 2 Materials and methods

### 2.1 Classification of mares

All animal procedures were completed in accordance with the Institutional Animal Care and Use (IACUC) of Colorado State University under the guidelines of the approved protocol #2024–5,699. Unless otherwise stated, all chemicals were purchased from Thermo Fisher Scientific (Waltham, MA, United States). Horses (*Equus caballus*) used in this study were mixed breed mares (11–25 years of age) ranging from 450 to 550 kg housed on pasture with hay *ad libitum*. Research and teaching mares that were available for use were screened for PPID based on endocrine testing performed in summer of 2024 and 2025 as previously described by Miller et al. (9). Peripheral blood was collected pre- and 10 min post-intravenous injection of thyrotropin-releasing hormone (TRH). In brief, 1.0 mg TRH (Wedgewood; Lakewood CO, United States) was administered to all mares. Samples were collected in glass ethyldiaminetetraacetic acid (EDTA) tubes and immediately placed on ice. Following centrifugation at 800 x g, plasma was separated and stored at −80 °C until analysis at a commercial laboratory (Cornell University Animal Health Diagnostic Center) as previously described by Adams et al. (17). ACTH>120 pg./mL following stimulation were defined as PPID, while animals with ACTH<60 pg./mL post-stim were defined as controls in accordance with previous publications while reflecting month of sampling (17). At least one clinical symptoms of PPID such as hirsutism, hypertrichosis, muscle atrophy, polyuria, polydipsia, and recurrent infections was noted in each animal enrolled. Group demographics are described in Table 1.

**Table 1:** Mean ± SEM mare age, circulating ACTH concentrations, and clinical symptoms.

Group	n	Age	ACTH pre-stim	ACTH post-stim	Clinical symptoms
Study 1 (Endometrium)					
Control	4	14.5 ± 0.5 yo^a^	24.8 ± 8.7 pg./mL^a^	90.1 ± 31.0 pg./mL^a^	0/4^a^
PPID	7	16.1 ± 0.7 yo^a^	51.9 ± 8.0 pg./mL^b^	381.6 ± 99.8 pg./mL^b^	7/7^b^ Hypertrichosis: 3/7 Muscle Atrophy: 3/7 Recurrent infections: 1/7
Study 2 (Follicular Fluid)					
Control	9	21.1 ± 1.2 yo^a^	24.6 ± 2.6 pg./mL^a^	47.2 ± 5.6 pg./mL^a^	0/9^a^
PPID	9	23.7 ± 0.6 yo^a^	190.6 ± 113.9 pg./mL^b^	554.6 ± 175.4 pg./mL^b^	9/9^b^ Hypertrichosis: 5/9 Muscle Atrophy: 2/9 Pendulous Abdomen: 2/9

PPID, Pars Pituitary Intermediate Dysfunction. ACTH, Adrenocorticotropic Hormone. ^a,b^indicates significant differences within columns for individual studies. *p* < 0.05.

### 2.2 Collection of endometrial tissue

In the northern hemisphere summer of 2024, 11 aged maiden mares were utilized to collect endometrial tissue (*n* = 7 PPID; *n* = 4 control). Reproductive cycles of all mares were followed via palpation and transrectal ultrasonography. Two endometrial biopsies were obtained with sterile alligator jaw biopsy forceps once mares were determined to be in diestrus (presence of a corpus luteum, toned cervix and lack of endometrial edema). Biopsies were placed in either RNALater or 10% formalin. Samples in RNALater were stored at 4°C for 24 h before being transferred to −20°C for long-term storage prior to RNA isolation. Samples in 10% formalin were stored at 4°C for 24 h before being transferred to methanol for long-term storage prior to paraffin embedding.

### 2.3 Collection of follicular fluid

In the northern hemisphere summer of 2025, follicular fluid was collected from the preovulatory follicle of a separate group of 17 mares (*n* = 7 PPID; *n* = 10 control) of similar ages and reproductive status (Table 1). Due to the mares being leased from external entities, complete reproductive histories were not available to the investigators. As a result, prior breeding performance, pregnancy outcomes, and historical reproductive management could not be fully documented for this study. Reproductive cycles were assessed using ultrasonography until a follicle of >33 mm and uterine edema consistent with estrus was observed. Follicle maturation was induced through the administration of histrelin (0.5 mg; IM; Doc Lane’s Pharmacy, Lexington KY). Approximately 18 h later, follicular fluid was collected under sedation using a transvaginal (TVA) guided technique, as previously described by Carnevale et al. (18). A 12-gage double-lumen oocyte aspiration needle (Cook Medical, Bloomington, IN, United States) was advanced through the needle guide encasing a transvaginal ultrasound transducer. The needly was advanced into the middle of the follicle antrum, and follicular fluid was collected into a conical tube using gentle suction to prevent blood contamination. Follicular fluid was aliquoted into 2 mL cryovials before storage at −80 °C until further processing.

### 2.4 Quantitative polymerase chain reaction analysis

Total RNA was extracted from 50 mg of endometrial tissue using TRIzol^®^ Reagent (Invitrogen, Carlsbad, CA, United States) as described by the manufacturer. Total RNA was precipitated using sodium acetate and isopropanol, resuspended in ddH_2_O and DNAse treated (DNA-free™, Applied Biosystems) and then analyzed for quantity and quality via a NanoDrop^®^ spectrophotometer (Thermo Scientific, Wilmington, DE, United States). RNA was reverse transcribed and qRT-PCR was performed as previously described by Fedorka et al. (19). Briefly, 1.5 μg of RNA in 41.5 μL ddH_2_0 was reverse transcribed using Promega reagents; 0.5 μL AMV Reverse Transcriptase, 16 μL 5x RT Buffer, 1 μL RNAsin®, 16 μL MgCl, 4 μL dNTP, and 1 μL Oligo(dT) Primer (Promega, Madison, WI, United States). Samples were incubated at 42 °C for 60 min followed by 95 °C for 5 min. Complimentary DNA (cDNA) was diluted 1:1 with ddH_2_0, and qPCR was performed using 4.5 μL of cDNA, 5 μL of Sensimix™ II (Bioline, Tauton, MA, United States) and 0.5 μL of a custom primer/probe set from Applied Biosystems. Primer sequences were designed using the TaqMan^®^ Gene Expression System (Thermo Fisher) and are described in Fedorka et al. (19). Reactions were performed in duplicate with beta-actin (ACTB) as the reference gene using the ViiA 7 Real-Time PCR System (Applied Biosystems, Grand Island, NY, United States). Samples were incubated at 95 °C for 10 min, followed by 45 cycles of 95 °C for 15 s and 60 °C for 60 s. Results were expressed as the mean -ΔCT.

### 2.5 Immunoassay

Serum and follicular fluid cytokines were analyzed using an equine-specific multiple sandwich immunoassay based on flowmetric MILLIPLEX MAP® technology in accord with the workflow previously published (20). This included IL-1β, IL-6, CXCL8, IL-10, and TNF. Samples were measured un-diluted. Standards for serum samples were prepared with serum matrix added, while follicular fluid standards were prepared without matrix added to all standards and quality controls, following the guidelines of the manufacturer. Antibody was washed prior to the addition of streptavidin. The means of intra-and inter-assay coefficients of variation were 2.7 and 3.7%, respectively. The detection level was defined as the signal-to-noise-ratio (limit of detection) divided by the square root of 2.

### 2.6 Immunohistochemistry

Immunohistochemical staining was performed to determine cell-specific protein labeling of lysozyme for leukocyte detection using a commercial laboratory (Colorado State University Veterinary Diagnostic Laboratory; Fort Collins, CO, United States). Sections of endometrium (5 μm) were mounted on positively-charged Superfrost Plus Microscope Slides (VWR, Radnor, PA) and processed using the Bond Polymer detection system [Leica Biosystems, Buffalo Grove, IL; described by Klein et al. (21)]. Lysozyme (1:100, rabbit polyclonal, #PA0391, Leica Biosystems) immunostaining was performed. Tissue sections were directly incubated with the polymer-labeled goat anti-rabbit IgG conjugated to HRP after primary antibody incubation. Finally, sections were counterstained with hematoxylin (2 min) and mounted using Richard-Allan Scientific Mounting Medium (ThermoFisher Scientific). Five high powered fields from each mare were examined by two blinded researchers under light microscopy at 40x.

### 2.7 Statistical analysis

Data were analyzed using SAS 9.4® (SAS Institute Inc., Cary, NC, United States). Data were assessed for normality using a Shapiro-Wilkes test and equal variances with a Bartlett’s test. The model was assessed using a general linear additive model, with disease as a fixed effect, and mare as a random effect. Comparisons were made using a Mann–Whitney T-test to compare transcript expression, cytokine production, and number of leukocytes in PPID and control groups. Additionally, a linear regression was utilized to assess the relationship between concentrations of ACTH post-TRH stimulation and cytokine expression. Significance was set to *p* < 0.05 with trends noted at *p* < 0.10. Data are presented as the median ± interquartile ranges when not normally distributed and mean ± standard error when normally distributed.

## 3 Results

### 3.1 Systemic inflammatory cytokine production

**Figure 1:**
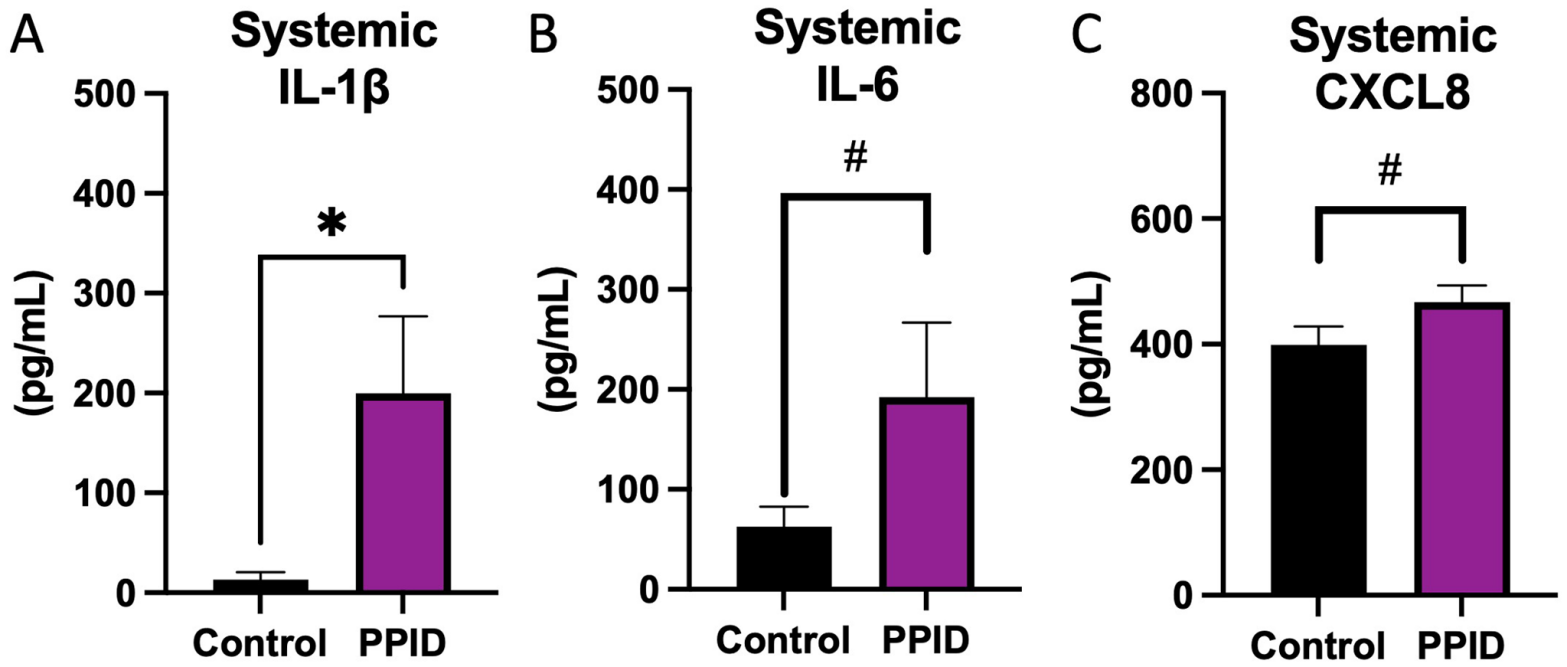
Systemic pro-inflammatory cytokine concentrations in control horses and horses diagnosed with PPID. **(A)** Circulating IL-1β, (B) IL-6, and **(C)** CXCL8 concentrations measured in serum samples from control horses and horses with pituitary pars intermedia dysfunction (PPID). Horses with PPID exhibited significantly greater systemic concentrations of IL-1β compared with controls **(A)**. Similarly, systemic IL-6 **(B)** and CXCL8 **(C)** concentrations were elevated in PPID horses relative to control animals. Data are presented as mean ± SEM and expressed in pg/mL. Statistical differences between groups are indicated above brackets (*p < 0.05, #*p* < 0.10).

Inflammatory cytokines were assessed in circulation of PPID and control animals to confirm system inflammation. While the majority of samples did not reach the limit of detection for IL-10 or TNF; an increase in IL-1β (*p* = 0.05; Figure 1A) alongside a tendency toward an increase in IL-6 (*p* = 0.06; Figure 1B) and CXCL8 (*p* = 0.08; Figure 1C) was noted in PPID mares when compared to controls.

### 3.2 mRNA expression of endometrial cytokines

**Figure 2:**
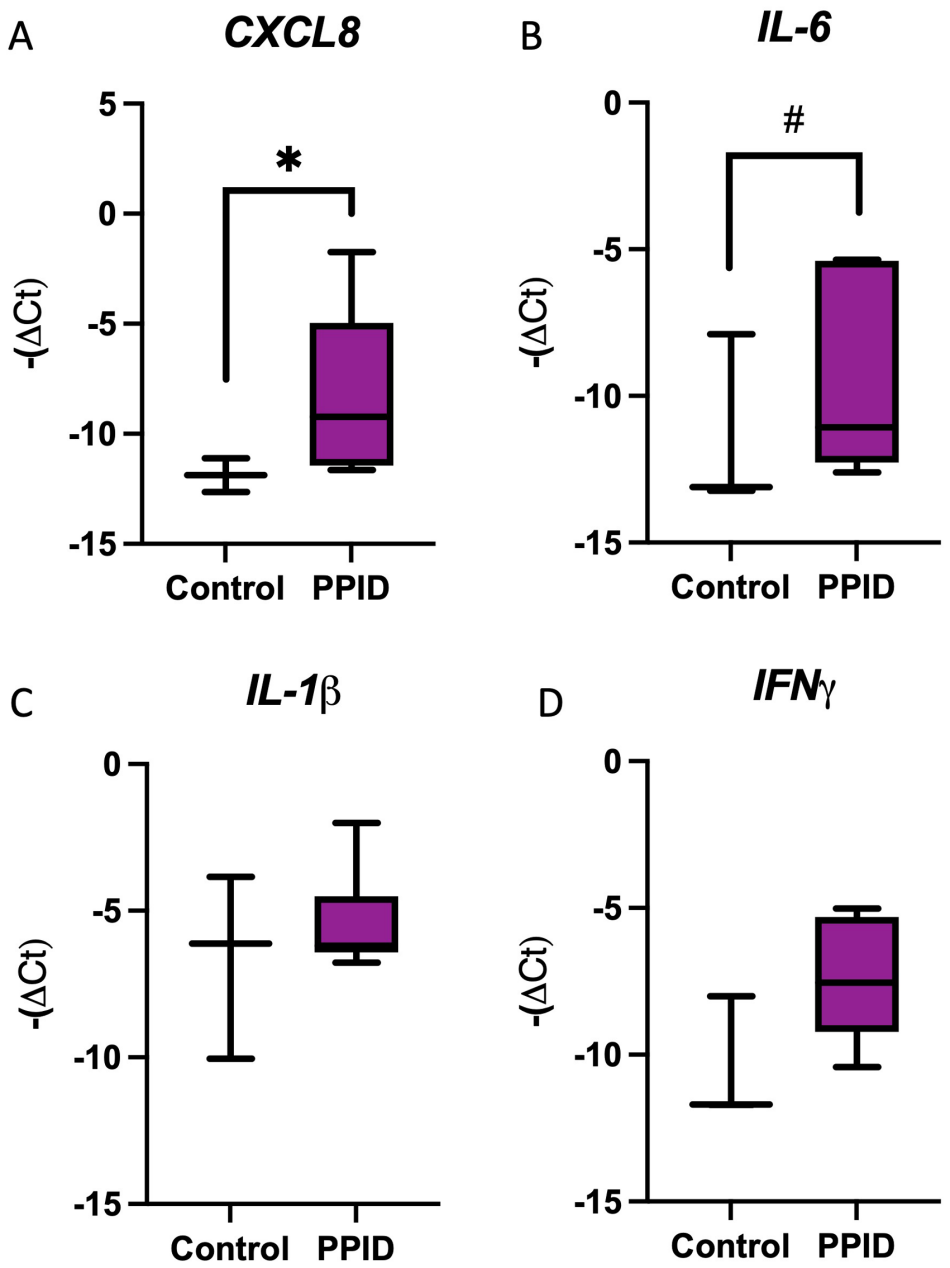
Endometrial cytokine gene expression in control horses and horses with pituitary pars intermedia dysfunction (PPID). Relative gene expression of pro-inflammatory cytokines in endometrial biopsy samples from control horses and horses diagnosed with PPID. Expression levels of **(A)** CXCL8, **(B)** IL-6, **(C)** IL-1β, and **(D)** IFNγ were quantified using qRT-PCR and are presented as −ΔCt values. Horses with PPID demonstrated significantly greater expression of CXCL8 **(A)** and IL-6 **(B)** compared with control horses. No significant differences were observed in *IL-1β* **(C)** or *IFNγ* **(D)** expression between groups. Data are presented as box-and-whisker plots showing median, interquartile range, and minimum–maximum values. Statistical significance is indicated above brackets (*p < 0.05, #*p* < 0.10).

The mRNA expression of key inflammatory cytokines was evaluated to assess if the presence of systemic inflammation coincided with endometrial inflammation. When assessing the endometrial expression of *CXCL8*, an increase was noted in PPID mares when compared to controls (*p* = 0.02; Figure 2A). Additionally, endometrial IL-6 tended to be greater in PPID mares when compared to controls (*p* = 0.09; Figure 2B). When compared to controls, gene expression in the endometrium of PPID mares for *IL-1β* (*p* = 0.73; Figure 2C), and *IFN-γ* (*p* = 0.49; Figure 2D) were similar.

**Figure 3:**
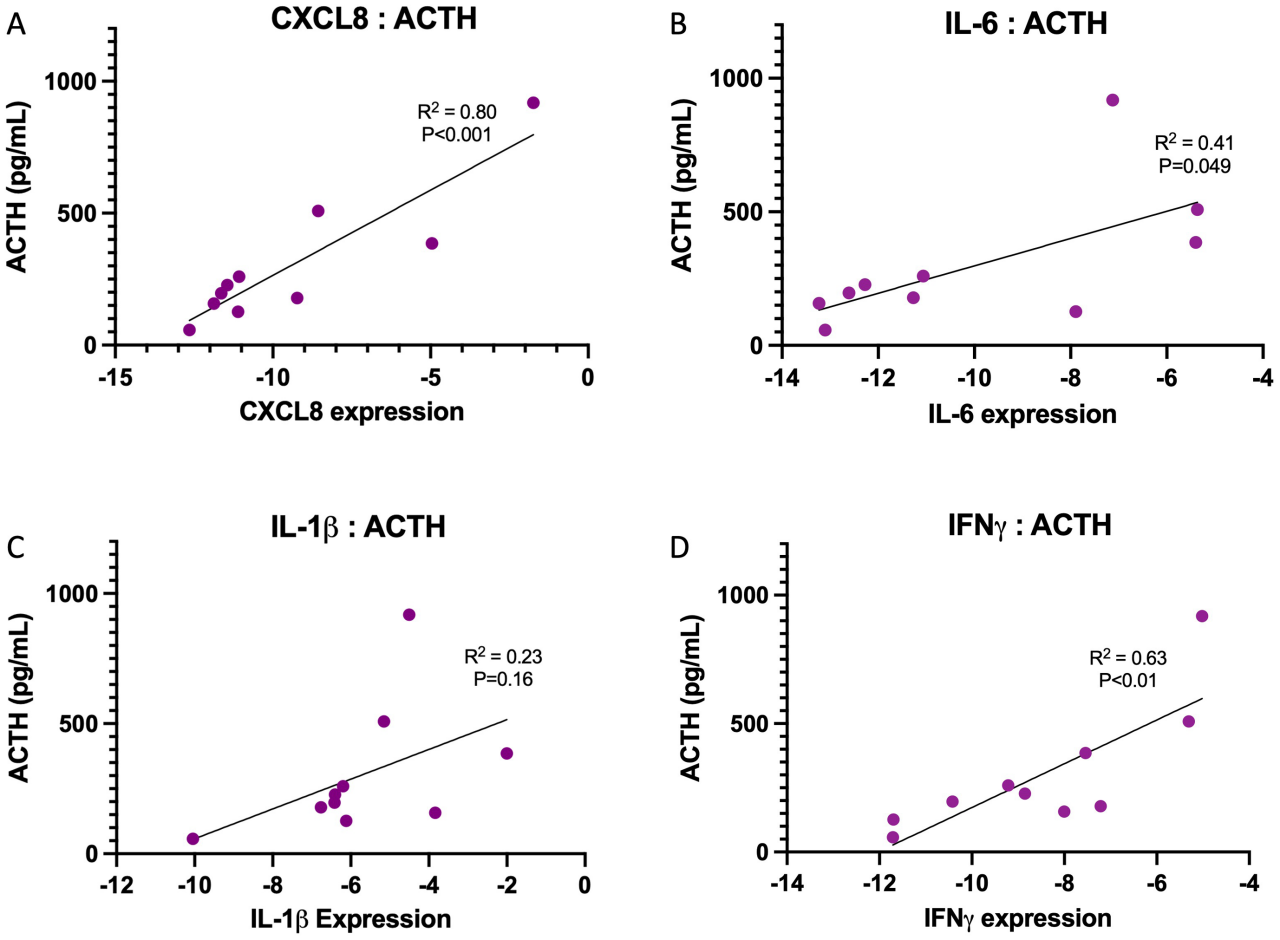
Associations between endometrial cytokine gene expression and circulating ACTH concentrations in horses. Linear regression analyses were performed to evaluate relationships between endometrial cytokine gene expression and plasma ACTH concentrations. **(A)** CXCL8 expression demonstrated a strong positive association with ACTH concentrations (R² = 0.80, *p* < 0.001). **(B)** IL-6 expression was moderately positively correlated with ACTH concentrations (R² = 0.41, *p* = 0.049). **(C)** IL-1β expression showed no significant association with ACTH concentrations (R² = 0.23, *p* = 0.16). **(D)** IFNγ expression was positively associated with ACTH concentrations (R² = 0.63, *p* < 0.01). Each point represents an individual horse. Cytokine expression values are presented as −ΔCt relative expression levels, and ACTH concentrations are expressed in pg/mL. Lines represent linear regression fits for each cytokine.

When assessing the correlation between endometrial gene expression of key cytokines and circulating ACTH after TRH-stimulation, a significant and positive correlation was noted between ACTH and *CXCL8* (*p* < 0.001; R^2^ = 0.80; Figure 3A). Weak but significant correlations were also noted when assessing the correlation between ACTH post-TRH stimulation and the endometrial expression of *IL-6* (*p* = 0.049; R^2^ = 0.41; Figure 3B) and *IFNγ* (*p* < 0.01; R^2^ = 0.63; Figure 3C). No correlation was noted between the endometrial expression of *IL-1β* and ACTH (*p* = 0.16; R^2^ = 0.23; Figure 3D).

### 3.3 Presence of endometrial leukocytes

**Figure 4:**
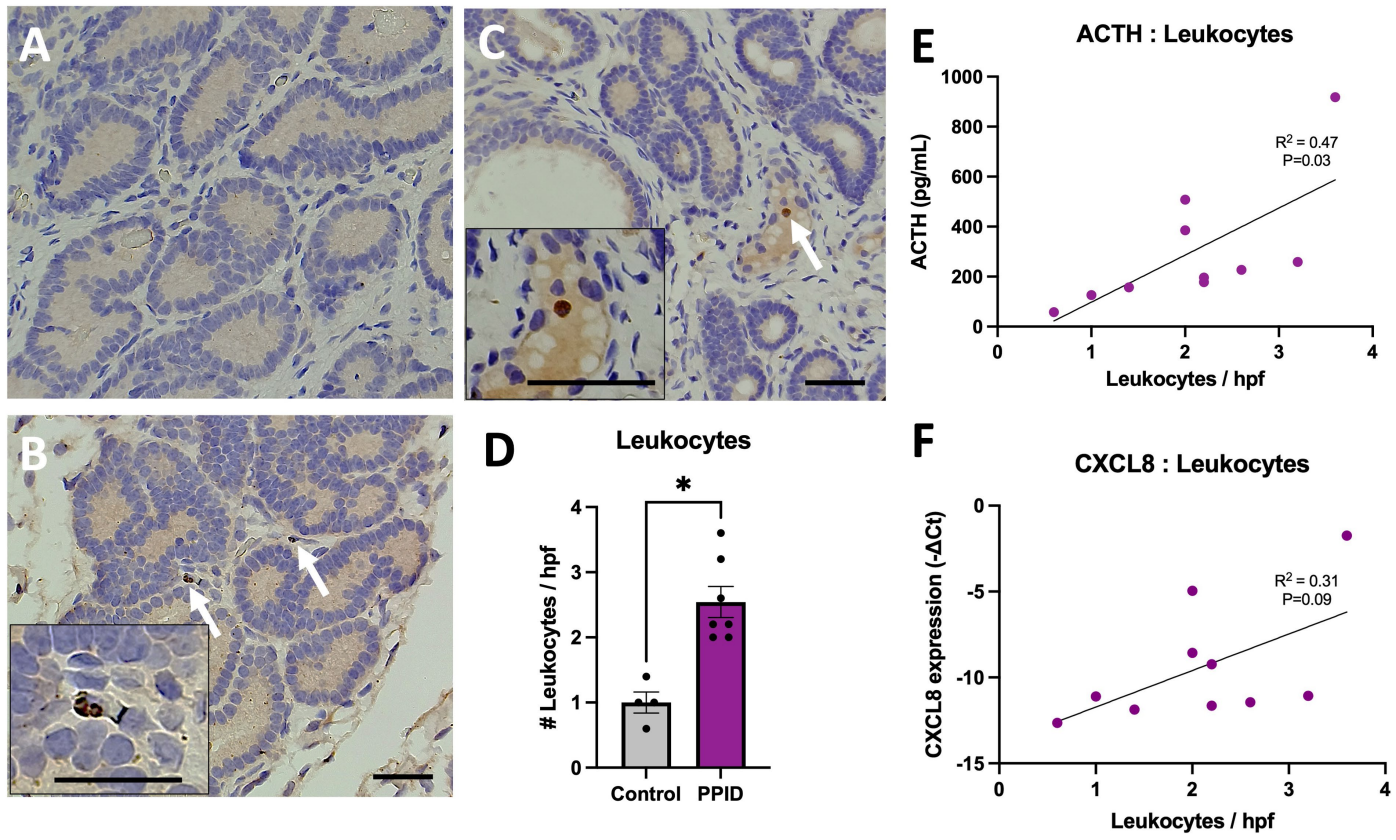
Leukocyte infiltration in the endometrium of control and PPID horses and associations with ACTH. Representative endometrial histologic sections from **(A)** control horses and **(B–C)** horses diagnosed with pituitary pars intermedia dysfunction (PPID). Arrows indicate leukocytes within the endometrial stroma. Insets show higher-magnification images highlighting individual leukocytes. Scale bars represent the indicated magnification. **(D)** Quantification of leukocytes per high-power field (hpf) revealed significantly greater leukocyte infiltration in PPID horses compared with control horses (*p < 0.05). **(E)** Linear regression analysis demonstrated a positive association between circulating ACTH concentrations and leukocyte infiltration within the endometrium (R² = 0.47, *p* = 0.03). **(F)** A positive trend was observed between endometrial CXCL8 expression and leukocyte infiltration (R² = 0.31, *p* = 0.09). Data are presented as mean ± SEM for leukocyte counts, with individual data points shown. Each point represents an individual horse. Cytokine expression values are presented as −ΔCt relative expression levels, and ACTH concentrations are expressed in pg/mL.

Due to the increase in mRNA expression of *CXCL8* in the PPID population, endometrial leukocytes were counted and found to be significantly higher in the endometrium of PPID mares when compared to controls (2.5 ± 0.2 vs. 1 ± 0.2; *p* = 0.03; Figure 4D). Neutrophils were primarily noted within glands (Figure 4B) while macrophages were observed within or near vessels (Figure 4C). Nornal healthy endometrium is represented as 4A. The number of leukocytes present in the endometrium were weakly correlated with *CXCL8* expression (*p* = 0.09; R^2^ = 0.31; Figure 4F), but the number of endometrial leukocytes with the concentration of circulating ACTH noted following TRH stimulation was significant (*p* = 0.03, R^2^ = 0.47; Figure 4E).

### 3.4 Follicular fluid cytokine production

**Figure 5:**
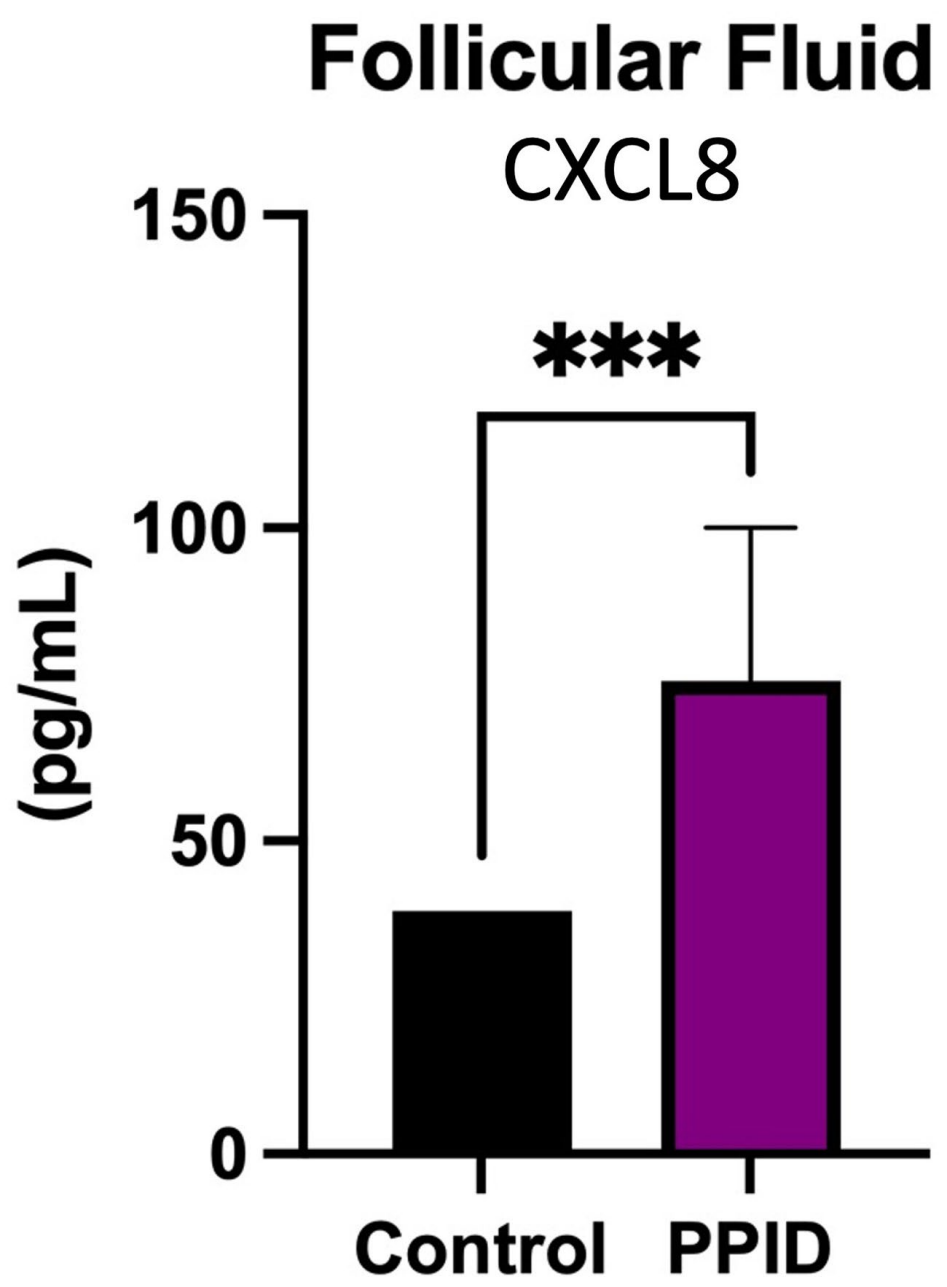
CXCL8 concentrations in follicular fluid of control and PPID mares. CXCL8 concentrations were quantified in follicular fluid collected from control mares and mares diagnosed with pituitary pars intermedia dysfunction (PPID). Follicular fluid from PPID mares exhibited significantly greater CXCL8 concentrations compared with control mares. Data are presented as mean ± SEM and expressed in pg/mL. Statistical significance is indicated above the bracket (***p < 0.001).

Pro-inflammatory cytokines assessed in the follicular fluid of PPID and control animals did not reach the limit of detection for IL-1β, IL-6, IL-10, or TNF; however, an increase in follicular fluid CXCL8 was found in the PPID population (*p* < 0.05) when compared to controls (Figure 5).

## 4 Discussion

Pituitary pars intermedia dysfunction (PPID) is of growing concern in the aged equine population due to its life-threatening comorbidities. Advancements in diagnostics, alongside improved owner awareness have led to an increase in disease diagnosis in younger aged animals, indicating a broad population impact (2, 22, 23). While studies have found PPID to greatly impact systemic endocrine and immune systems (7–9), its impact on the reproductive tract is poorly understood, but associated with subfertility (24). In the present study, we noted increased inflammation within the reproductive tract of PPID animals. This included an upregulation of various inflammatory cytokines that coincided with increased endometrial leukocyte presence. Additionally, this was noted at the protein level within follicular fluid of the preovulatory follicle in PPID mares. These findings may elucidate the pathophysiology behind the subfertility noted in this population of animals.

Formerly known as equine Cushing’s disease, PPID is a neurodegenerative disorder that is believed to affect a large portion of the aged equine population (1). It has been shown that animals affected with PPID have persistent elevated inflammation, which may lead to laminitis and secondary infections in addition to prolonged healing time from primary infections (1, 6). This altered inflammatory profile was also noted within the present study, as mares with PPID were found to have increased pro-inflammatory cytokine production systemically. Additionally, PPID-associated inflammation was noted in the reproductive tract, including both the diestrus endometrium and pre-ovulatory follicular fluid. PPID mares experienced elevated diestrus endometrial *CXCL8*, with a trend toward an increase in expression of *IL-6*. Additionally, this study found a strong positive correlation between the endometrial expression of *CXCL8* and the concentration of ACTH found in circulation following stimulation with TRH. This relationship was also described by Zak et al., where ACTH concentrations in PPID animals were found to have a significant and positive correlation with circulating CXCL8 concentrations (25). The primary function of CXCL8 is chemotaxis of leukocytes toward sites of inflammation, which is crucial for response to pathogens and foreign particles (10). Without disease or disorder, chronic leukocytosis will perturb homeostasis of the tissue. In other species, an increase in CXCL8 production is noted in many autoimmune and inflammatory diseases, including chronic obstructive pulmonary disease (COPD) (26), psoriasis (27), rheumatoid arthritis (28), and inflammatory bowel disease (29). In the human reproductive tract, chronic elevation of CXCL8 has been associated with endometriosis (30), which serves as a biomarker for the severity of disease (31). The impact of persistent endometrial CXCL8 in the horse within this population on reproductive performance deserves future attention.

An inflammatory cascade is activated following the deposition of anything foreign into the uterine lumen. This is initiated by the detection of pattern recognition receptors (PRP), and the production of pro-inflammatory mediators IL-1β and CXCL8 by epithelial cells (10). Chemotaxis of leukocytes from the stroma of the endometrium into the uterine lumen occurs within 30 min following insemination, with leukocyte clearance occurring within 48 h (14). This inflammatory mechanism is referred to as breeding-induced endometritis. In the uterus, this inflammation is essential for the clearance of spermatozoa, seminal plasma, and pathogens following insemination (32). However, inflammation must resolve before the embryo migrates from the oviduct to the uterine lumen, which occurs at roughly 5.5 days after fertilization (33). Therefore, the immune response of the uterus must act in two partsboth in the detection and attack of potentially pathogenic molecules, in addition to the tolerance and —support of the developing semi-allogeneic embryo (14, 34–36). Mares that do not resolve this uterine inflammation in a timely manner are considered susceptible to the disease of persistent breeding-induced endometritis (PBIE), and are sub-fertile (32). Similar to PPID, PBIE is primarily noted in aged animals (37). In the present study, the number of leukocytes was significantly greater in PPID mares than in controls. This increase in leukocytes was positively correlated to ACTH concentrations noted following stimulation with TRH. Elevated ACTH has been found to be negatively associated with fertility in the mare, and this may be explained by the increase in neutrophil chemotaxis due to heightened ACTH (38). While the increase in leukocytes within the endometrium is not surprising considering the heightened *CXCL8* expression (39–41), it is disconcerting that the diestrus endometrium of the PPID mare is inflamed. In estrus, the normal equine endometrium has a heightened numbers of leukocytes, but this is drastically reduced in diestrus (42), primarily due to the anti-inflammatory effects of progesterone in preparation for embryo receptivity and implantation (43). While the specific day of diestrus was not known within this study, an influx of leukocytes into the endometrium at this time would be detrimental to embryo survival and potentially explains aspects of subfertility anecdotally noted in the PPID population. Future studies would include breeding trials of PPID animals alongside exploration of treatments that may improve fertility to further elucidate this link.

The current study also noted a trend toward an increase in the endometrial expression of *IL-6* in the PPID population. Additionally, a weak interaction between endometrial expression of *IL-6* and ACTH was found. This is in contrast to the findings of Zak et al., where no significant correlations were noted between systemic cytokines and ACTH, including IL-1β, IL-6, IFNγ, or TNF (25). It should be noted that the present study controlled for the phase of the estrous cycle, as all endometrial samples were obtained when mares were in diestrus while follicular fluid was collected in estrus. It has been described that stage of the estrous cycle, and the steroid hormones prominent within each stage, will impact the immune system - both within reproductive tissues in addition to systemically (44, 45). Estrogens are predominantly pro-inflammatory (46, 47), while progesterone activates anti-inflammatory aspects of immunity (43, 48). Therefore, it is notable that *CXCL8* and *IL-6* were elevated in the diestrus endometrium, which was under the influence of anti-inflammatory progesterone. The prior studies on PPID were not focused on reproductive parameters, so cycle stage was not controlled for, which may explain the discrepancies in *IL-6* expression or relationship to ACTH.

While an increase in IL-1β, IL-6, and CXCL8 was found systemically in the PPID mare, only CXCL8 was found to increase in the follicular fluid of the preovulatory follicle. The increase in CXCL8 is intriguing, as PPID mares experience an increase in anovulatory follicles (49). Inflammation is crucial for ovulation success, as the pre-ovulatory follicle experiences a surge in pro-inflammatory mediators (cytokines, prostaglandins, and chemokines) which recruit immune cells to the follicular wall (12). These immune cells release enzymes, proteins, and reactive oxygen species (ROS) that promote follicular wall remodeling, cumulus expansion, and oocyte release during ovulation (50). Simultaneously, the increase in prostaglandins increases vascular permeability and smooth muscle contractions which will allow for rupture of the follicular wall (51). CXCL8 is a crucial mediator of this, as it is responsible for the recruitment of neutrophils to sites of inflammation. Crucial mediators of ovulation, these cells are luteinizing hormone (LH)-responsive and act as endocrine and inflammatory mediators due to their ability to release both PGF_2_α and ROS (52). Chronic inflammation in the follicular environment has been associated with ovarian dysfunction in women (53). This is believed to be due to persistent oxidative stress alongside the low antioxidant capacity of follicular fluid, which impedes oocyte quality and release (54, 55). Therefore, the impact of elevated CXCL8 in the PPID population may partially explain ovulatory failure, and potentially have downstream impacts on oocyte maturation, fertility, and embryonic failure. Unfortunately, the clinical ramifications of this increase in CXCL8 could not be confirmed within the confines of this study, but is an obvious next step.

Limitations of this study include a small sample size, delayed sampling time, and non-specific cell labeling. Eleven animals were included in the investigations into endometrial health due to limitations on herd size, while a larger sample size was utilized for follicular fluid. This study was powered to detect biologically meaningful differences in key inflammatory endpoints, particularly CXCL8, based on prior equine PPID literature. Sample sizes reflect the limited availability of well-characterized PPID mares and are consistent with comparable mechanistic studies in equine reproductive immunology. Additionally, it was difficult to identify age-matched control without elevated ACTH. While smaller sample sizes increase the risk of Type II error for subtle effects, significant differences and strong correlations observed indicate adequate power for the primary outcomes. This study would have been improved by immunophenotyping specific cell populations within the endometrium, but lack of antibody validation within the equine model with increased specificity prohibited this factor. Finally, additional reproductive tissue and function need to be evaluated, including the cervix, ovarian tissue, and myometrial function, and the clinical impacts of each assessed. Considerable research is warranted and still required to fully elucidate the impact of PPID on reproductive function.

In conclusion, PPID and its associated endocrinopathies appear to increase inflammation both systemically in addition to the reproductive tract. This was noted as an increase in endometrial expression of inflammatory mediators *CXCL8* and *IL-6*, both of which were both positively correlated with the amount of ACTH noted in circulation following stimulation with TRH. The increase in *CXCL8* is associated with an increased presence of leukocytes within the endometrium. Additionally, an increase in CXCL8 was noted both in circulation and within follicular fluid, highlighting the systemic nature of this pathology. Future research is required to evaluate the fertility of PPID animals, in addition to evaluating other aspects of the reproductive tract, including oocyte quality and ovarian physiology.

## Data Availability

The raw data supporting the conclusions of this article will be made available by the authors, without undue reservation.
