# Supplementary figures and images for: Impact of pituitary pars intermedia dysfunction on inflammation within the equine reproductive tract of the mare
Source: Front Vet Sci. 2026 Mar 26;13:1758501. doi: 10.3389/fvets.2026.1758501 (PMC13064447; doi:10.3389/fvets.2026.1758501)

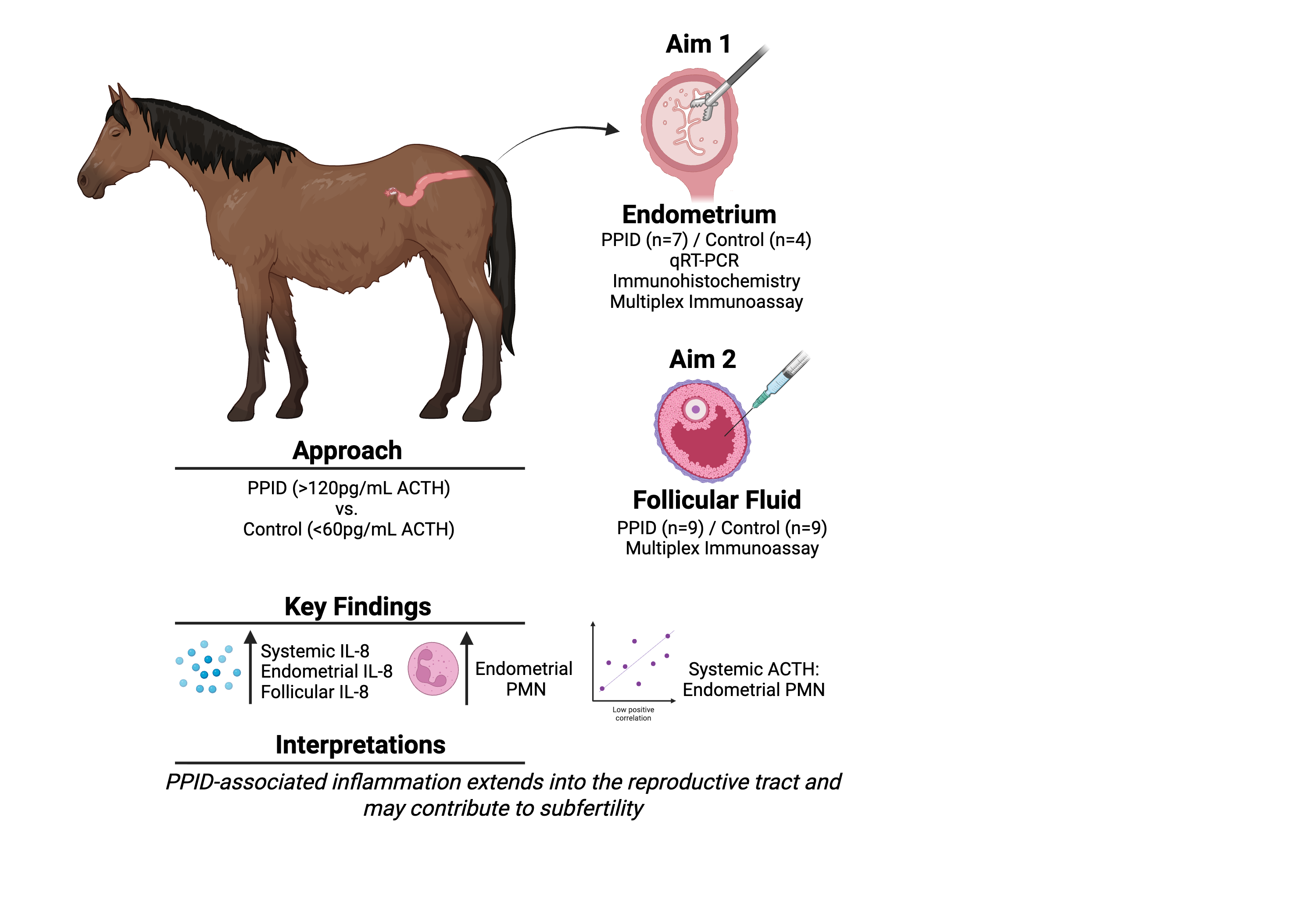

Supplement: Supplementary file 1 [file Image_1.PNG]
